# Clinical management of metastatic gastric tumors: a case report of right popliteal leiomyosarcoma metastasizing to the stomach and literature review

**DOI:** 10.3389/fonc.2026.1805796

**Published:** 2026-05-04

**Authors:** Ping Chen, Tun Yue, Dan Shi, Xiaofei Bi, Wei Zhang, Zecheng Wu, Chengting Zheng

**Affiliations:** 1 Department of Gastroenterology, Chongqing University Three Gorges Hospital, Chongqing University, Chongqing, China; 2 Department of Pathology, Chongqing University Three Gorges Hospital, Chongqing University, Chongqing, China; 3 Department of Gastrointestinal Surgery, Chongqing University Three Gorges Hospital, Chongqing University, Chongqing, China; 4 Department of Nephrology, Chongqing University Three Gorges Hospital, Chongqing University, Chongqing, China

**Keywords:** case report, gastric malignancies, hemorrhage, leiomyosarcoma, metastasis

## Abstract

Gastric metastases are rare, accounting for only 0.2%–0.7% of gastric malignancies. Gastric metastasis from primary popliteal fossa leiomyosarcoma (LMS) has not been previously reported. We report a 60-year-old female who presented with hematemesis and severe anemia, with a past medical history of right popliteal fossa LMS resection. Imaging and endoscopy revealed a bleeding nodular lesion in the gastric body. Pathology and immunohistochemistry (SMA+/Desmin+, CD117–/DOG-1–) confirmed metastatic LMS to the stomach, with suspected pancreatic and bone metastases. The patient underwent emergency embolization for active bleeding, followed by laparoscopic partial gastrectomy and systemic chemotherapy. At the 9-month follow-up, she remained in good general condition. This case highlights that in patients with a history of malignancy presenting with gastrointestinal bleeding, gastric metastasis should be considered, and multidisciplinary management combined with surgery and systemic therapy can achieve favorable outcomes.

## Introduction

Gastric cancer is among the most common malignancies of the digestive system, accounting for approximately 5.6% of new cancer cases worldwide (1). Although the stomach is rich in blood vessels and lymph nodes, metastatic cancer to the stomach is relatively rare, constituting only 0.2–0.7% of all gastric malignancies in clinical series (2), with an incidence of 5.4% reported in an autopsy study of 6380 cases (3).

The most common primary sites for gastric metastases include malignant melanoma and carcinomas of the breast and lung. Metastasis occurs less frequently from cancers of the thyroid, esophagus, and kidney (4–6). Soft tissue leiomyosarcoma (LMS) originating in the lower extremities is exceedingly rare, with an estimated incidence of <1% among extremity sarcomas. Only 12 cases of LMS in the popliteal fossa have been reported in the past decade (7). To our knowledge, there are no published reports of popliteal LMS metastasizing to the stomach. Our center recently managed a case of gastric metastasis from a primary right popliteal fossa LMS. This unique case is reported here, along with a systematic review of the literature on gastric metastases.

## Case presentation

A 60-year-old female presented with a ten-hour history of hematemesis and fatigue. Her medical history included a mass in the right popliteal fossa present for over 11 years. Four years ago, the patient noticed that the mass was growing and underwent excision at a local hospital. At the time of surgery, the patient had no pain, difficulty walking, or other discomfort. The postoperative specimen was about 4*3cm, sent for pathological examination, and confirmed a diagnosis of “right popliteal fossa leiomyosarcoma.” To prevent recurrence, the patient came to our hospital and underwent 30 fractions of local radiotherapy and three cycles of chemotherapy (Ifosfamide + Epirubicin). Subsequently, the patient did not adhere to regular follow-up or further treatment. Her past medical history also included hypertension, type 2 diabetes mellitus, and hepatitis B surface antigen carrier status, managed with medication.

**Figure 1:**
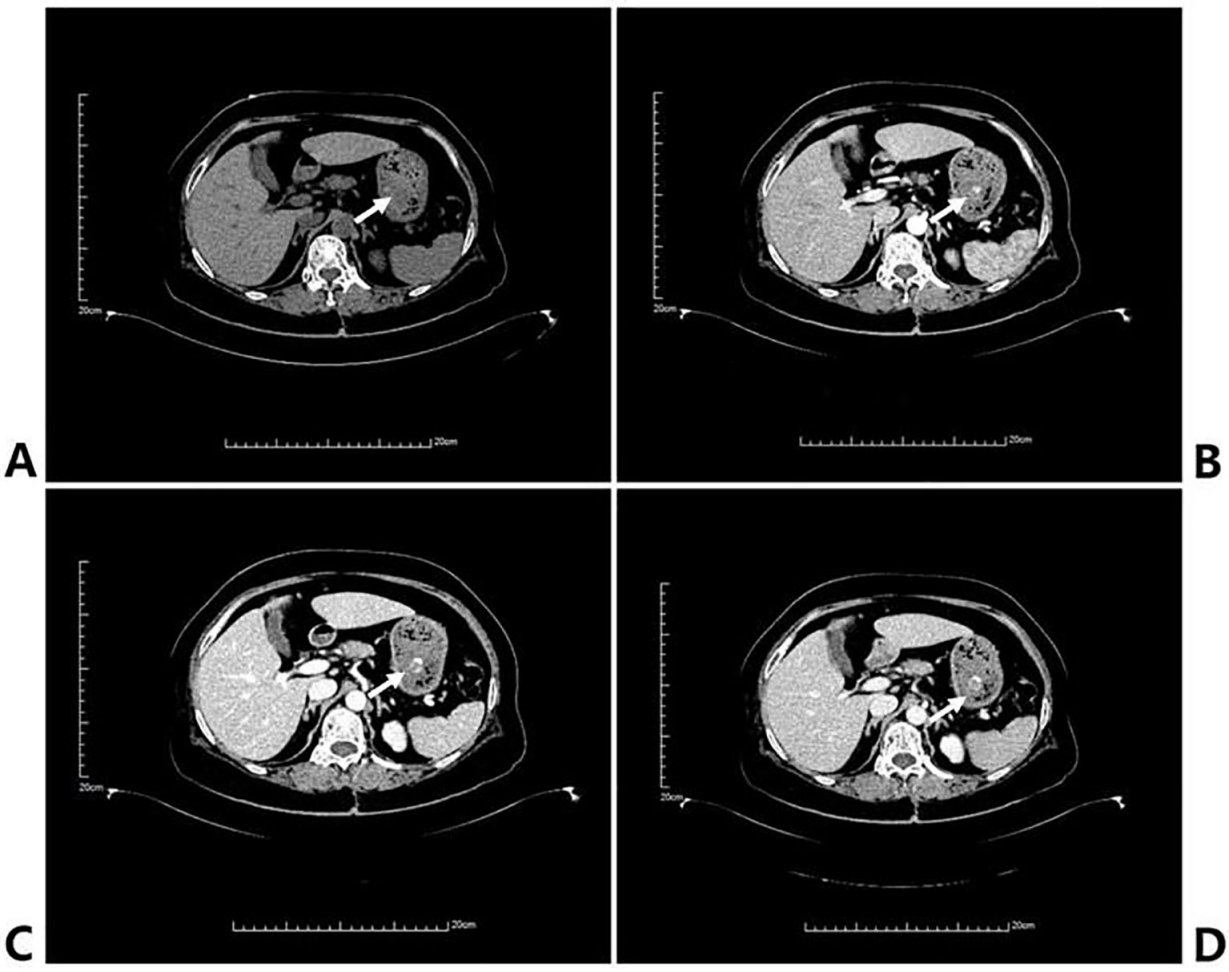
The manifestations of CT scans in different stages of the metastatic tumors in the stomach. **(A)** CT plain scan shows a locally nodular protrusion in the gastric body wall, appearing as a slightly hypodense shadow. **(B–D)** The arterial, portal venous, and delayed phases demonstrate heterogeneous, significant nodular enhancement.

Upon current admission, she reported dizziness, palpitations, and profuse diaphoresis accompanying the hematemesis. She denies any discomfort, such as abdominal pain, bloating, poor appetite, acid reflux, fainting, or altered consciousness. Physical examination was significant only for pallor and a surgical scar in the right popliteal fossa. No masses were palpated in the right popliteal fossa or anywhere else in the body. Emergency laboratory studies revealed severe anemia: RBC 1.79×10^12/L, Hb 55 g/L, Hct 16.2%, consistent with normocytic anemia. Contrast-enhanced abdominal CT demonstrated abundant gastric content and a pedunculated intraluminal nodular lesion, approximately 1.2 cm in diameter, which showed intense blood-pool-like enhanced post-contrast (**Figure 1**).

**Figure 2:**
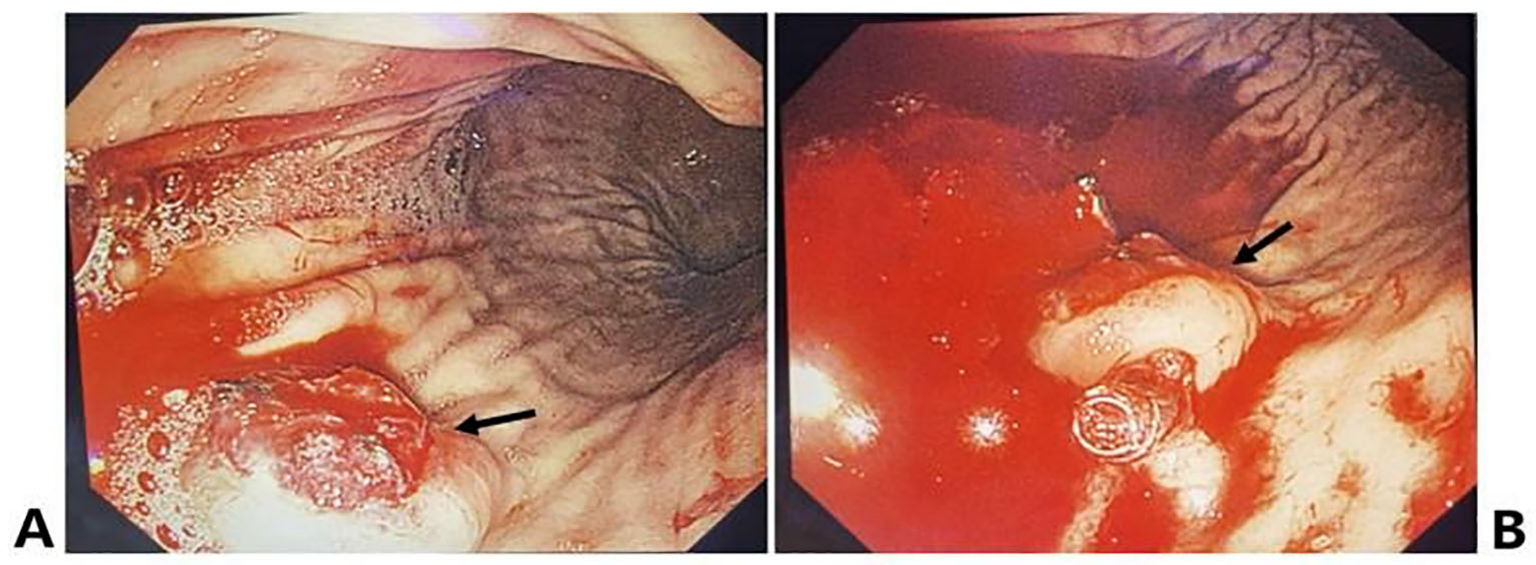
Endoscopic appearance of metastatic tumors in the stomach. **(A)** A nodular, protruding lesion (1.5 cm) with active bleeding and a fragile surface was identified on the greater curvature of the upper gastric body. **(B)** Endoscopic titanium clips were applied around the lesion to mark it.

Initial management included acid suppression, gastric cytoprotection, volume expansion, hemostatic agents, and blood transfusion. Approximately 40 minutes post-admission, she experienced a massive hematemesis of approximately 1000 ml, accompanied by mental confusion and hemodynamic instability (BP 80-85/45–49 mmHg, HR 105–115 bpm). Resuscitation continued with aggressive transfusion, fluid resuscitation, and vasopressor support (Dopamine and Metaraminol). Emergency esophagogastroduodenoscopy (EGD) revealed a large volume of fresh blood and clots within the gastric lumen. A nodular, protruding lesion (1.5 cm) with active bleeding and a fragile surface was identified on the greater curvature of the upper gastric body. Endoscopic titanium clips were applied around the lesion for marking (**Figure 2**). For the acute active bleeding, no biopsy was performed; the endoscopic impression was bleeding gastric carcinoma. For acute hemostasis, urgent digital subtraction angiography (DSA) was performed, revealing abnormal contrast staining and extravasation from the distal left gastric artery, consistent with tumor-associated hemorrhage. Successful transarterial embolization of the left gastric artery was performed, achieving hemostasis. A subsequent PET-CT scan demonstrated: 1) Focal nodular thickening of the gastric wall at the greater curvature with increased FDG uptake, suggestive of a neoplastic lesion; 2) Nodules in the pancreatic body and tail with increased FDG uptake, suggestive of metastatic disease; 3) An osteolytic lesion in the right scapula with increased FDG uptake, suggestive of bone metastasis; 4) No evidence of local tumor recurrence in the right popliteal fossa post-resection.

**Figure 3:**
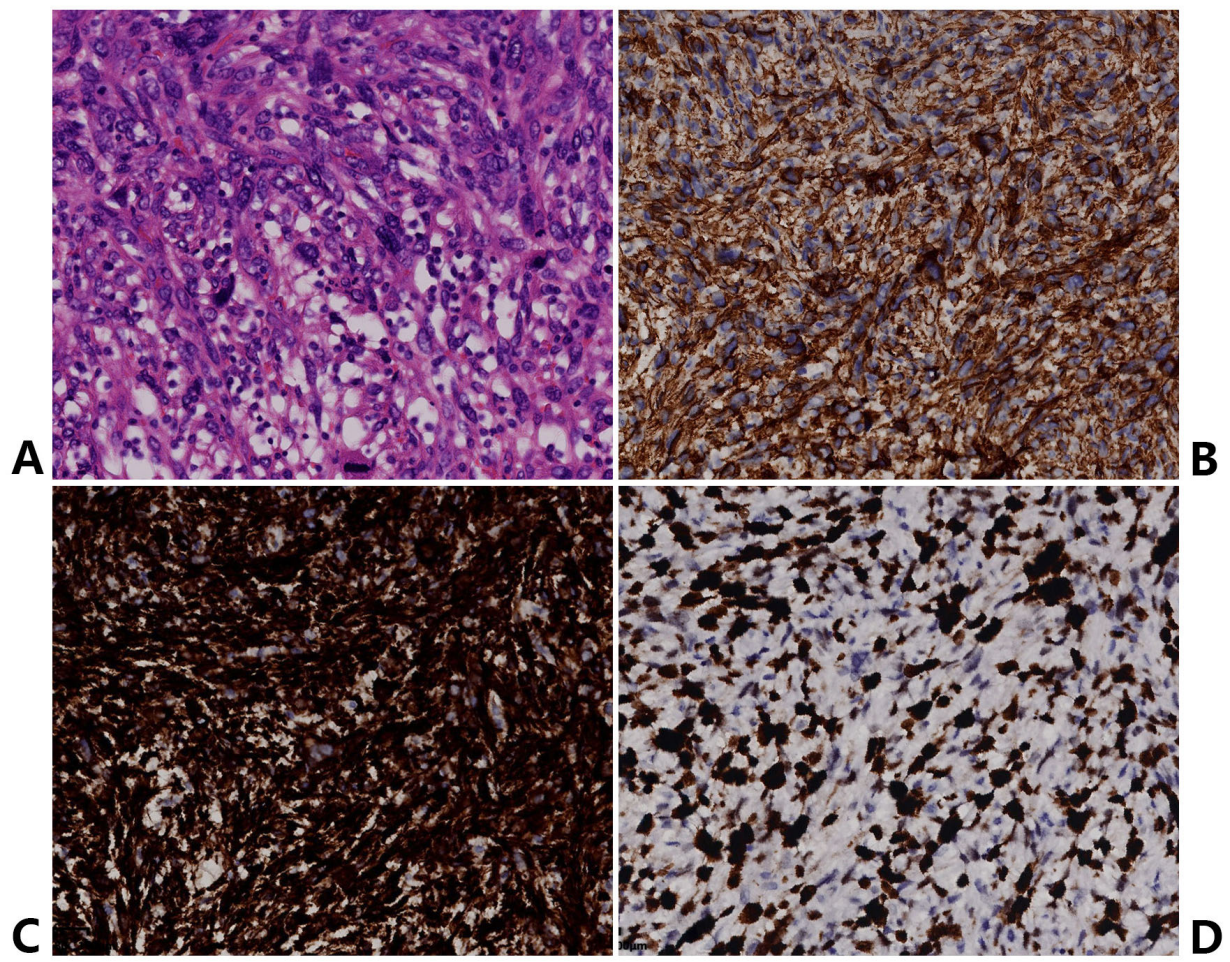
Histopathological and immunohistochemical features of the gastric metastasis originating from a right popliteal leiomyosarcoma. **(A)** Tumor cells are spindle-shaped with abundant eosinophilic cytoplasm, showing marked nuclear atypia, coarse chromatin, and prominent nucleoli (H&E, ×200). **(B)** SMA exhibits diffuse cytoplasmic positivity in tumor cells, confirming smooth muscle differentiation (IHC, ×200). **(C)** Desmin shows strong, diffuse cytoplasmic expression, further supporting myogenic differentiation (IHC, ×200). **(D)** Ki-67 labeling index is approximately 80%, reflecting high proliferative activity and aggressive tumor behavior (IHC, ×200).

After the bleeding stopped, to further clarify the diagnosis, a deep biopsy of the gastric mass was performed to obtain pathological tissue, and the pathology indicated that the cells are spindle-shaped with abundant eosinophilic cytoplasm, showing marked nuclear atypia, coarse chromatin, and prominent nucleoli, consistent with leiomyosarcoma (**Figure 3A**). Cellular morphology suggested a spindle cell tumor. To confirm the diagnosis, immunohistochemical staining was performed, revealing the following results: SMA (+), Desmin (+), CD34 (+), SDHB (+), H3K27me3 (+), Ki-67 index 80% (**Figures 3B–D**), and negative for CD117, DOG-1, S-100, PCK, and P40. In this immunohistochemical profile, positivity for SMA and Desmin supports a smooth muscle origin and is the key evidence for the diagnosis of leiomyosarcoma. Negativity for CD117 and DOG-1 excludes gastrointestinal stromal tumor (GIST). Negativity for S-100 excludes melanoma and schwannoma. Negativity for PCK and P40 excludes carcinoma (including squamous cell carcinoma and adenocarcinoma). Positivity for H3K27me3 excludes malignant peripheral nerve sheath tumor (MPNST). Positivity for SDHB excludes SDH-deficient GIST. A Ki-67 proliferation index of 80% indicates a high-grade leiomyosarcoma. Finally, this immunohistochemical profile supports the diagnosis of gastric leiomyosarcoma. Combining medical history and PET-CT results, we diagnosed the gastric tumor as a metastatic tumor caused by leiomyosarcoma of the right popliteal fossa.

Due to the high risk of recurrent hemorrhage and following multidisciplinary team (MDT) discussion, the patient underwent laparoscopic partial gastrectomy. Intraoperative exploration revealed a mobile tumor measuring approximately 1.5 cm. A Mindray disposable linear cutting stapler was used to detach and excise the tumor from the gastric wall. Postoperative pathology confirmed the preoperative diagnosis of leiomyosarcoma. Exploration of the pancreas revealed a tumor approximately 3 cm in diameter, with a hard texture and poor mobility. No obvious tumors were found in the peritoneum, omentum, spleen, liver, colon, pelvic cavity, or abdominal cavity. The definitive diagnosis was metastatic gastric leiomyosarcoma (primary: right popliteal fossa) complicated by acute hemorrhage, with suspected pancreatic and osseous metastases. The patient received adjuvant systemic chemotherapy with Gemcitabine (1.5g) and Dacarbazine(250mg) for 3 cycles. Follow-up assessments to date indicate a good general condition with no major complications.

## Discussion

Metastatic involvement of the stomach is uncommon, accounting for only 0.2–0.7% of gastric malignancies (2). The most frequent primary sites are breast cancer, melanoma, and lung cancer. Rare metastases, such as sarcomatoid carcinoma of the jejunum, choriocarcinoma, and seminoma, have also been reported (8–10). Soft tissue LMS of the lower extremities is exceedingly rare, representing <1% of extremity sarcomas, with only 12 cases of popliteal fossa LMS reported in the last decade (7). To our knowledge, metastasis from popliteal LMS to the stomach has not been previously documented. Despite its rarity, the stomach should be considered a potential site for metastasis, especially as the global cancer burden increases. Clinicians must maintain vigilance for gastric metastases in patients with a history of extra-gastric malignancies.

The mechanisms of gastric metastasis are not fully elucidated and likely vary by primary tumor. Proposed pathways include peritoneal dissemination, hematogenous spread, lymphatic dissemination, and direct invasion (2). Hematogenous and lymphatic routes are thought to be predominant. Endoscopically, approximately 50% of metastatic gastric tumors present as submucosal tumor-like lesions, while about 40% resemble primary gastric cancer (3). This pattern is consistent with hematogenous or lymphatic spread seeding the submucosal layer.

Clinical features of gastric metastases are non-specific. Patients may present with pain, nausea, vomiting, or bleeding (11). Iron-deficiency anemia and guaiac-positive stools are common (12). Gastric bleeding was the most common clinical symptom, occurring in approximately 32.4% of patients, while some patients exhibited no symptoms (13). While EGD can detect abnormalities, there are no pathognomonic features. Lesions are variably described as masses, nodules, mucosal irregularities, ulcers, or polyps. Metastases tend to involve the proximal stomach (fundus and body) more often than the distal stomach (antrum and pylorus; 54.1% vs. 29.3%), and tumors involving overlapping sites were less frequent (6). They can be single or multiple and often exhibit central umbilication, likely due to rapid growth and necrosis. In our case, the initial presentation was a life-threatening hemorrhage. Endoscopy revealed a bleeding nodular lesion, which could not be distinguished from primary adenocarcinoma or a bleeding submucosal tumor based on morphology alone.

Histopathology remains the diagnostic cornerstone. Gastric metastases typically retain the morphological and immunophenotypic characteristics of the primary tumor, a key feature for distinguishing them from primary gastric cancer. Specific IHC markers aid identification: e.g., HMB45/MelanA/S-100 for melanoma; TTF-1/CD56/CgA for small cell lung cancer; GPC-3/Sall4 for hepatocellular carcinoma; ER/PR/Her-2 for breast cancer; and CDX2/CK20/SATB2 for colorectal adenocarcinoma (14). Our case was diagnosed based on cytomorphology and an IHC profile (SMA+, Desmin+, SDHB+, H3K27me3+, CD34+, Ki-67 index 80%), consistent with LMS and matching the primary popliteal tumor. However, tumor heterogeneity can pose diagnostic challenges. Immunophenotypic loss during metastasis has been reported, such as in some gastric metastases from hepatocellular carcinoma (15). Furthermore, discrepancies between primary and metastatic sites can occur, as noted in some breast cancer metastases where ER/PR may be lost, while other targets (e.g., PDL1, HER2) may be expressed (14). Koji S et al. reported a prostate cancer metastasis to the stomach where serum PSA was normal, but IHC for prostate-specific markers (AMACR, PSAP) was positive (16). In our case, the identical IHC profile to the known primary facilitated the diagnosis.

The prognosis for patients with gastric metastasis is generally poor. According to the literature, about half of patients with gastric metastasis also have metastatic lesions in other organs. The mean time from the diagnosis of gastric metastasis to death is approximately 4.75 months (17), with a reported median survival of 18.0 months after the primary cancer diagnosis, and only 3.0 months after the diagnosis of gastric metastasis. Patients with a solitary lesion demonstrated more prolonged median survival after the diagnosis of metastatic cancer than patients with multiple lesions (13). Management options include surgery, radiotherapy, chemotherapy, and supportive care. Surgical intervention is typically reserved for complications such as bleeding, obstruction, or perforation. As gastric metastasis usually indicates advanced disease, systemic therapy tailored to the primary tumor is the mainstay of treatment (5, 18). Patient performance status, treatment history, and extent of metastatic disease significantly influence therapeutic decisions. For patients with solitary metastases and no widespread disease, aggressive local therapy, including surgery, may offer long-term survival (19, 20). Both Y. I. Kim and Ga Hee Kim reported that patients benefited from surgical treatment and achieved longer survival (11, 13). Nanako et al. reported that submucosal invasion was observed in 40.9% of gastric metastases measuring 10 mm or less. To achieve R0 resection, full-thickness surgical resection is preferred. In the future, endoscopic full-thickness resection may be used to achieve R0 resection when gastric metastasis is resectable (10, 20). In our case, we performed a complete surgical resection of the bleeding gastric metastasis followed by systemic chemotherapy (Gemcitabine and Dacarbazine) based on the primary LMS protocol. The patient tolerated treatment well with no significant complications at the 9-month follow-up.

Looking back at this case overall, the patient presented us with two major difficulties. The first difficulty was diagnosis. Based solely on clinical manifestations and endoscopic findings, it was difficult to distinguish or even suspect that the massive bleeding was caused by metastatic gastric cancer. A definitive diagnosis could only be made by combining the patient’s medical history and pathological reports. This also reminds clinicians and endoscopists that although metastatic gastric cancer is rare, whenever a patient has a history of malignancy, we should consider the possibility of metastatic gastric cancer. The second difficulty was the choice of treatment. Given that distant metastasis had already occurred, systemic chemotherapy was undoubtedly indicated. For the treatment of the local gastric mass, we performed surgery only after a multidisciplinary team (MDT) discussion. The reasons for choosing surgery for the gastric mass were as follows (1): the patient was in generally good condition and could tolerate the surgery (2); the patient had a strong personal desire for surgery (3); to prevent recurrent life-threatening massive bleeding; and (4) the hope that resection of the metastatic lesion might lead to longer survival. During subsequent follow-up, no new lesions were found in the patient’s stomach, and the pancreatic and bone metastases had significantly improved. The patient is still alive and well. Overall, we achieved a favorable outcome.

## Conclusion

Metastatic tumors to the stomach, which can originate from diverse primary sites, are associated with a poor prognosis. A meticulous clinical history remains paramount for diagnosis. Pathological cytology and immunohistochemistry are the gold standard for diagnosis. Metastatic gastric cancer indicates that distant metastasis has occurred; systemic chemotherapy is the cornerstone of all treatments. Whether surgery is beneficial requires a comprehensive assessment. Multidisciplinary team (MDT) management is crucial for optimizing patient outcomes. Further case accumulation and research are needed to refine the diagnostic and therapeutic strategies for this challenging clinical entity.

## Data Availability

The original contributions presented in the study are included in the article/**Supplementary Material**. Further inquiries can be directed to the corresponding authors.
