# Supplementary material for: Clinical management of metastatic gastric tumors: a case report of right popliteal leiomyosarcoma metastasizing to the stomach and literature review
Source: Front Oncol. 2026 May 4;16:1805796. doi: 10.3389/fonc.2026.1805796 (PMC13180607; doi:10.3389/fonc.2026.1805796)
Supplement: Supplementary file 2 [file DataSheet2.docx]

Supplementary Material

## Supplementary Figures


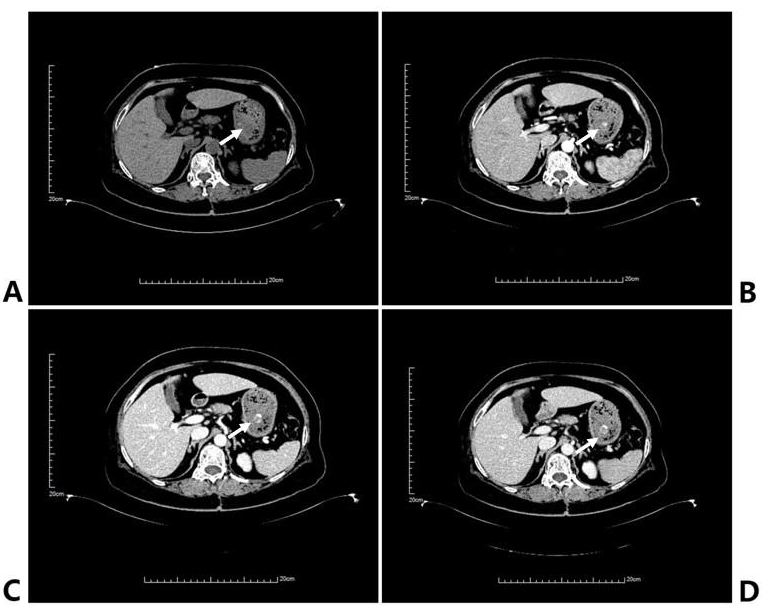


## Supplementary Figure 1. The manifestations of CT scans in different stages of the metastatic tumors in the stomach.(A)CT plain scan shows a locally nodular protrusion in the gastric body wall, appearing as a slightly hypodense shadow.(B-D)The arterial, portal venous, and delayed phases demonstrate heterogeneous, significant nodular enhancement.


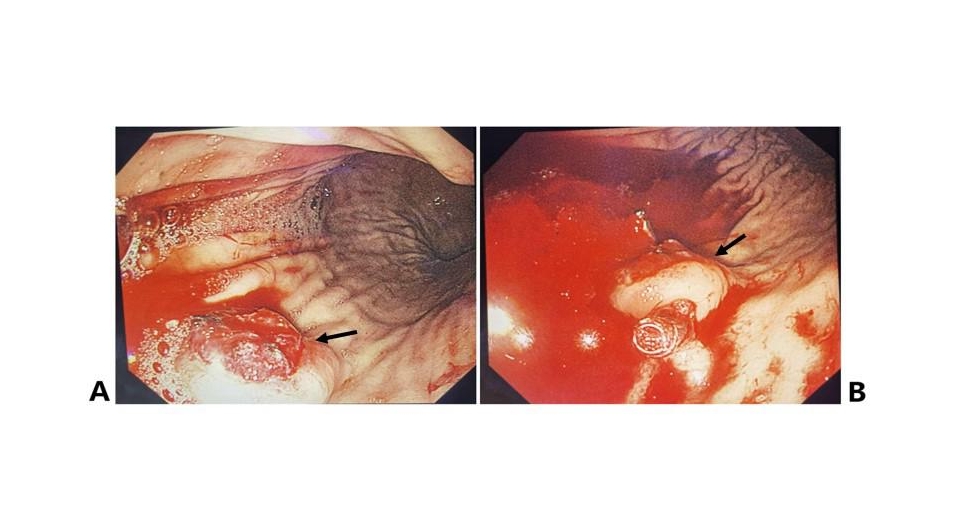


**Supplementary Figure 2.** Endoscopic appearance of metastatic tumors in the stomach.(A)A nodular, protruding lesion(1.5cm)with active bleeding and a fragile surface was identified on the greater curvature of the upper gastric body.(B)Endoscopic titanium clips were placed around the lesion for marking.


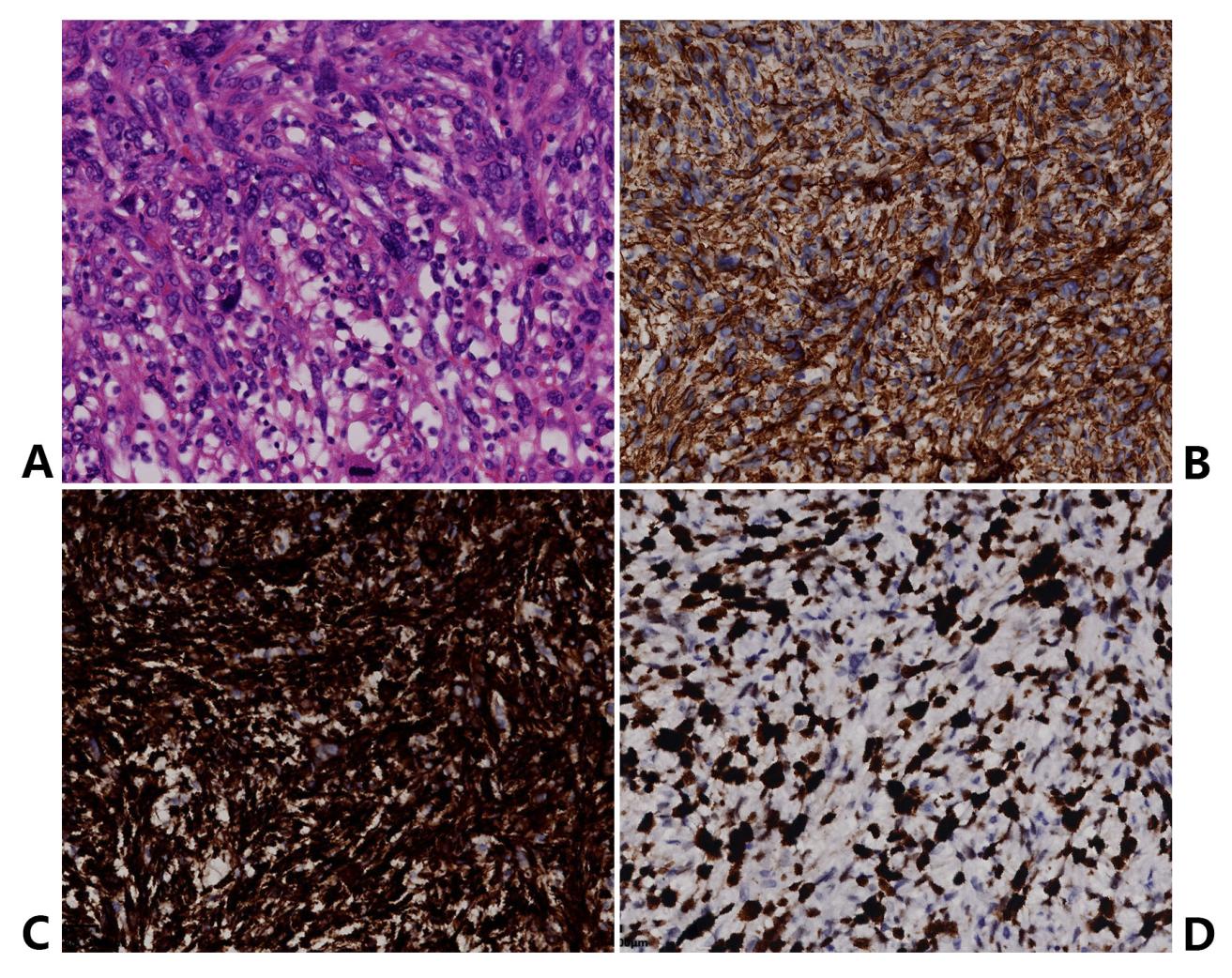


**Supplementary Figure 3.** Histopathological and immunohistochemical features of the gastric metastasis originating from a right popliteal leiomyosarcoma.(A)Tumor cells are spindle-shaped with abundant eosinophilic cytoplasm, showing marked nuclear atypia, coarse chromatin, and prominent nucleoli(H&E, ×200).(B)SMA exhibits diffuse cytoplasmic positivity in tumor cells, confirming smooth muscle differentiation (IHC, ×200).(C)Desmin displays strong and diffuse cytoplasmic expression, further supporting myogenic differentiation (IHC, ×200).(D)Ki-67 labeling index is approximately 80%, reflecting high proliferative activity and aggressive tumor behavior(IHC, ×200).
